# Feasibility of bedside portable MRI in neonates and children during ECLS

**DOI:** 10.1186/s13054-023-04416-7

**Published:** 2023-04-04

**Authors:** Hemmen Sabir, Florian Kipfmueller, Soyhan Bagci, Till Dresbach, Tamara Grass, Patrizia Nitsch-Felsecker, Christos Pantazis, Joachim Schmitt, Lukas Schroeder, Andreas Mueller

**Affiliations:** Department of Neonatology and Pediatric Intensive Care, Children’s Hospital, University of Bonn, Venusberg-Campus 1, 53127 Bonn, Germany

**Keywords:** Newborn, Brain imaging, ECLS, Critical care medicine

## Abstract

Magnetic resonance imaging (MRI) is the preferred neuroimaging technique in pediatric patients. However, in neonates and instable pediatric patients accessibility to MRI is often not feasible due to instability of patients and equipment not being feasible for MRI. Low-field MRI has been shown to be a feasible neuroimaging tool in pediatric patients. We present the first four patients receiving bedside high-quality MRI during ECLS treatment. We show that it is safe and feasible to perform bedside MRI in this patient population. This opens the route to additional treatment decisions and may guide optimized treatment in these patients.

## Introduction

Neuroimaging during modern intensive care medicine often remains a challenge. Especially in pediatric patients easily accessible availability of high-quality brain imaging remains an often unbeatable hurdle [1]. Neonatal ultrasound is the standard technique in preterm and term neonates to detect common brain injuries, as intraventricular hemorrhage, hypoxic-ischemic brain injury and stroke [2]. However, it depends on an open fontanel and deep brain structures can often not be visualized. In addition, the quality of the imaging technique is examiner dependent. Computerized tomography (CT) is a valuable broadly available imaging tool. It still requires transport of the patient, but image acquisition is fast and acute detoriations can be quickly diagnosed. Though, because of the potential for increased radiation exposure to children undergoing these scans, pediatric CT has been stated as being a public health concern and should be avoided in this patient population [3]. Magnetic resonance imaging (MRI) is the preferred imaging technique for neuroimaging in pediatrics. However, its daily accessibility often remains a challenge. In patients undergoing invasive procedures during their intensive care stay, as well as in extremely premature infants, MRI is often not available and patient transport is not feasible due to the severity of the underlying illness [4].

Low-field portable MRI (pMRI) has been reported to be a feasible and diagnostic accurate method to image the brain on the bedside [5]. One first study in neonates and pediatric patients has shown that pathologies detected by pMRI images correlate well with pathologies detected on conventional MRI [6]. However, the study had not included severely sick neonates or children during invasive procedures as extracorporeal life support (ECLS). To date, only one study has reported three adult patients with femoral cannulation, who have been examined with a pMRI system during ECLS treatment [7]. As it was shown to be feasible and safe, we aimed to investigate whether it was feasible and safe to image newborns and pediatric patients, often being cannulated with jugular cannulas, during treatment taking pMRI to the next feasibility level.

## Methods

This is an investigator-initiated cohort study. All patients were examined after consent was obtained from a legally authorized representative. Decision to perform pMRI was based on an individual option due to non-available access to alternative imaging options and individual clinical decision due to necessity of neuroimaging.

### Study population

This study included neonates and pediatric patients (< 18 years) during ECLS treatment.

### Study procedure

All patients were studied during ECLS treatment on the bedside in the patient’s room. All patients were mechanically ventilated and cannulated with a jugular ECLS cannula during pMRI imaging. Before initiation of the first scan, the ECLS cannula was tested for magnetic response and it was found to be non-present in the pMRI system. After parental consent, patients were moved into the 64mT Swoop® MRI imaging system (Hyperfine, Guilford, CT, USA; hardware version 1.7, software version 8.4.0) with the help of three individuals (one intensivist and two nurses). One additional intensivist monitored the situation and gave feedback to the team while moving the patient, if applicable. The patient’s head was fixed with inflatable pads, preventing head motion during imaging. Vital signs (heart rate, breathing rate, transcutaneous oxygen saturation, invasive blood pressure) were monitored continuously during the study. Axial T1 and T2 weighted images were obtained from every patient. After completion of the MRI, the patient was moved back to the bed by the same team as previously described.

### Outcomes

Primary outcome was feasiblity and safety to complete the pMRI and to receive good quality MRI images without adverse event (1. Changes in vital signs ± 20%; 2. Changes in ECLS flow rate ± 20%; 3. Changes in ECLS cannula position). Secondary outcome was time to move the patient in and out of the pMRI and time to obtain T1 and T2 weighted images. In addition, nursing staff satisfaction during the procedure was recorded (1 = fully satisfied; 5 = not satisfied).

## Results

All patient characteristics are presented in Table 1. In brief, this study presents the first four pediatric patients with veno-venous (VV, *n* = 3) or veno-arterial (VA, *n* = 1) ECLS undergoing pMRI during ECLS treatment.

**Table 1:** Patient characteristics

Patient	ECLS device	ECLS cannulation (VV or VA)	ECLS indication	Changes in vital signs	Changes in ECLS flow rate	Time to move the patient into/out of the pMRI (minutes)	MRI time to obtain the images (minutes)	Staff satisfaction (1 = fully satisfied; 5 = not satisfied
1	Medos Deltastream circuit with a DP3 pump, and a hilite 800 LT oxygenator system, Xenios AG, Aachen, Germany	VV (13 French jugular double lumen cannula; Avalon Elite, Getinge, Rastatt, Germany)	Lung hypoplasia due to multicystic dysplastic kidneys	None	None	25/15	35	2
2	Medos Deltastream circuit with a DP3 pump, and a hilite 2400 LT oxygenator system, Xenios AG, Aachen, Germany	VV (14 French jugular and 14 French femoral single lumen cannula; Medtronic, Minneapolis, MN, USA)	ARDS due to Respiratory syncytial virus	None	None	18/10	25	1
3	Medos Deltastream circuit with a DP3 pump, and a hilite 7000 LT oxygenator system, Xenios AG, Aachen, Germany	VV (17 French jugular and 17 French femoral single lumen cannula; Medtronic, Minneapolis, MN, USA)	ARDS due to Influenza A	None	None	12/8	18	1
4	Medos Deltastream circuit with a DP3 pump, and a hilite 7000 LT oxygenator system, Xenios AG, Aachen, Germany	VA (15 French jugular and 19 French femoral venous and 15 French femoral arterial single lumen cannula; Medtronic, Minneapolis, MN, USA)	Cardiogenic shock due to Parvovirus B19 associated cardiomyopathy	None	None	15/10	22	1

Patients were treated with either veno-venous (VV) or veno-arterial (VA) ECLS

*ARDS* acute respiratory distress syndrome

### Patient details

All patient details are presented in Table 1. Times to move the patient and time to obtain the images were acceptable (Table 1). Patient 1 (term newborn; 2.8 kg) was treated with VV ECLS and cannulated with one double lumen cannula (13 French) into the right internal jugular vein. pMRI was performed on day 6 as intracranial hemorrhage was suspected by ultrasound and to investigate congenital central nervous system congenital malformations. Due to the size of the patient, the patient was moved into the pMRI including his chest. Patient 2 (2 years, 15 kg) was treated with VV ECLS and cannulated with one single lumen cannula into the right internal jugular vein (14 French) and one single lumen cannula into the left internal femoral vein (14 French). pMRI was performed on day 9 after initiation of ECLS treatment due to changes in hemoglobin levels and near infrared spectroscopy (NIRS) signal to exclude intracranial hemorrhage. Patient 3 (10 years, 30 kg) was treated with VV ECLS and cannulated with one single lumen cannula into the right internal jugular vein (17 French) and one single lumen cannula into the left internal femoral vein (17 French). pMRI was performed on day 9 after initiation of ECLS treatment due to changes in NIRS values and coma without sedation to exclude intracranial hemorrhage or ischemia. Patient 4 (9 years, 35 kg) was treated with VVA ECLS and cannulated with one single lumen cannula into the right internal jugular vein (15 French), one single lumen cannula into the left femoral vein (19 French), and one single lumen cannula into the right femoral artery (15 French). pMRI was performed on day 23 after initiation of ECLS treatment due to changes in NIRS values and focal seizures.

### Outcomes

**Fig. 1:**
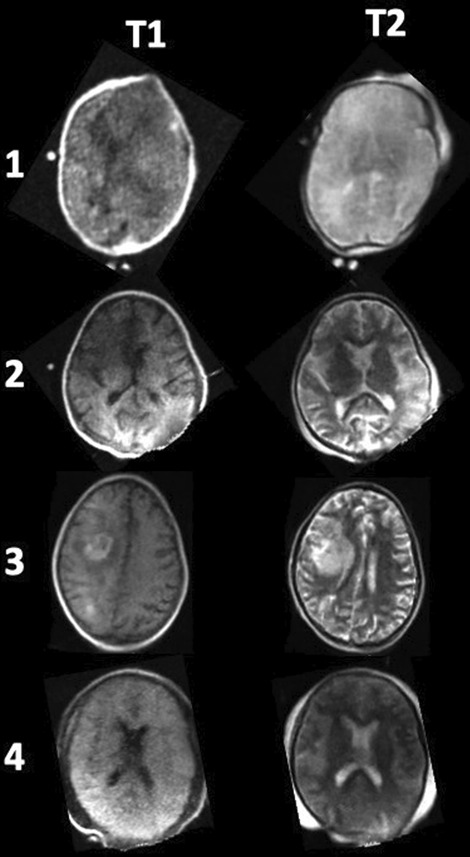
Representing T1 and T2-weighted images from the four patients receiving pMRI during ECLS treatment. In Patient 3 pMRI showed right hemispheric intracerebral hemorrhage. All other patients did not have signs of hemorrhage or stroke. Acquisition times: T1 (5m35s) and T2 (5m50s)

Primary outcome was achieved in all 4 patients without adverse events. T1 and T2 weighted images were obtained in each patient (Fig. 1), and no significant changes in vital signs were obtained during movement of patient or during neuroimaging. Jugular cannula position was controlled using echocardiography before and after neuroimaging. No changes were seen due to motion of the patient into the pMRI or out of the pMRI and cannula position remained safe. Time to move the patient into and out of the pMRI was between 8 and 25 min and staff was satisfied with the procedure of moving the patient and obtaining neuroimages (Table 1). No deleterious effects of the pMRI magnet on electrical equipment in the intensive care unit were noticed.

## Discussion

MRI is the gold standard neuroimaging technique in pediatric patients, but it requires MRI compatible equipment and monitoring. We present the first 4 pediatric intensive care patients receiving high-quality MRI during ECLS therapy on the bedside. We obtained images in all patients without any side effects. Images were obtained within an acceptable time and satisfaction of nursing staff, caring for the individual patient, was high. The special aspect of our patients is that all were cannulated with a jugular ECLS cannula while being scanned. The jugular ECLS cannula did not show any interference with the magnetic field, and noise was not present in the individual images. As we show feasibility and safety performing pMRI in pediatric patients while ECLS treatment, this opens a new opportunity to improve treatment strategies and provides evidence for clinical decisions in these patients. So far, NIRS and amplitude-integrated electroencephalography (aEEG) have been used for neuromonitoring [8]. However, both techniques have their weakness. Feasibility of pMRI could open a new field of neurocritical care and guide treatments for neuroprotection in intensive care patients.

It has previously been shown that pMRI was feasible in neonates during intensive care treatment [6]. In adults pMRI is feasible and safe and has 80% sensitivity and 97% specificity detect intracranial hemorrhage [9]. In adult patients pMRI has been performed during ECLS treatment [7]. In contrast to our study, all adult patients were cannulated in the femoral artery or vein, which provided better opportunities to move the patient into the pMRI system. We show that this is also feasible and safe with jugular cannulation.

One limitation of pMRI is image quality, the lack of important sequences for acute phases following brain injuries (e.g., diffusion-weighted images) and the potential to underdiagnose small hemorrhages or white matter injuries. As shown, the improvement of the pMRI system and new imaging sequences are currently developed [10]. As the cost of the pMRI system is much lower than a conventional MRI system and it does not require special building constructions, pMRI could be used in the future as a prognostic biomarker of long-term outcome in the pediatric population.

## Conclusion

Neonatal and pediatric patients can safely be moved into the pMRI system while being cannulated with a jugular cannula and treated with ECLS. This opens the route to additional treatment decisions and guides optimized treatment in these patients.

## Data Availability

All data can be accessed via the corresponding author.
